# α_2_- and β_2_-Adrenoreceptor-Mediated Efficacy of the Atypical Antidepressant Agomelatine Combined With Gabapentin to Suppress Allodynia in Neuropathic Rats With Ligated Infraorbital or Sciatic Nerve

**DOI:** 10.3389/fphar.2018.00587

**Published:** 2018-06-07

**Authors:** Saïd M’Dahoma, Matthieu Poitevin, Eric Dabala, Hugo Payan, Cecilia Gabriel, Elisabeth Mocaër, Sylvie Bourgoin, Michel Hamon

**Affiliations:** ^1^INSERM U894, Centre de Psychiatrie et Neurosciences, Paris, France; ^2^Institut de Recherches Internationales Servier, Suresnes, France

**Keywords:** chronic constriction injury, sciatic nerve, infraorbital nerve, neuropathic rats, mechanical allodynia, agomelatine, gabapentin, α_2_- and β_2_-adrenoreceptors

## Abstract

Previous data showed that neuropathic pain induced by mechanical lesion of peripheral nerves has specific characteristics and responds differently to alleviating drugs at cephalic versus extracephalic level. This is especially true for tricyclic antidepressants currently used for alleviating neuropathic pain in humans which are less effective against cephalic neuropathic pain. Whether this also applies to the antidepressant agomelatine, with its unique pharmacological properties as MT_1_/MT_2_ melatonin receptor agonist and 5-HT_2B_/5-HT_2C_ serotonin receptor antagonist, has been investigated in two rat models of neuropathic pain. Acute treatments were performed 2 weeks after unilateral chronic constriction (ligation) injury to the sciatic nerve (CCI-SN) or the infraorbital nerve (CCI-ION), when maximal mechanical allodynia had developed in ipsilateral hindpaw or vibrissal pad, respectively, in Sprague–Dawley male rats. Although agomelatine (45 mg/kg i.p.) alone was inactive, co-treatment with gabapentin, at an essentially ineffective dose (50 mg/kg i.p.) on its own, produced marked anti-allodynic effects, especially in CCI-ION rats. In both CCI-SN and CCI-ION models, suppression of mechanical allodynia by ‘agomelatine + gabapentin’ could be partially mimicked by the combination of 5-HT_2C_ antagonist (SB 242084) + gabapentin, but not by melatonin or 5-HT_2B_ antagonist (RS 127445, LY 266097), alone or combined with gabapentin. In contrast, pretreatment by idazoxan, propranolol or the β_2_ antagonist ICI 118551 markedly inhibited the anti-allodynic effect of ‘agomelatine + gabapentin’ in both CCI-SN and CCI-ION rats, whereas pretreatment by the MT_1_/MT_2_ receptor antagonist S22153 was inactive. Altogether these data indicate that ‘agomelatine + gabapentin’ is a potent anti-allodynic combination at both cephalic and extra-cephalic levels, whose action implicates α_2_- and β_2_-adrenoreceptor-mediated noradrenergic neurotransmission.

## Introduction

Among antidepressants currently used as first line treatment of chronic neuropathic pain, tricyclics, such as amitriptyline, and mixed inhibitors of noradrenaline (NA) and serotonin (5-HT) re-uptake, such as duloxetine and venlafaxine, are the most frequently prescribed. However, the resulting pain alleviation is limited as only one patient out of 4–6 treated by these drugs has a pain reduction of at least 30% (Finnerup et al., 2015). Furthermore, both tricyclics and mixed NA/5-HT reuptake inhibitors produce adverse side effects which can be poorly tolerated and lead to drop out. Recently, agomelatine, a novel antidepressant whose mechanism of action is markedly distinct from those of tricyclics and mixed NA/5-HT reuptake inhibitors, has also been found to exert anti-allodynic and anti-hyperalgesic effects in validated rodent models of neuropathic pain (M’Dahoma et al., 2015; Uçel et al., 2015; Aydin et al., 2016; Chenaf et al., 2017). Agomelatine is a mixed agonist at MT_1_ and MT_2_ melatonin (MT) receptors and an antagonist at 5-HT_2B_ and 5-HT_2C_ serotonin receptors with negligible affinity for the other 5-HT receptor types (Millan et al., 2003; Guardiola-Lemaitre et al., 2014). Extensive binding studies also showed that agomelatine has no affinity (Ki > 10 μM) for the specific NA, 5-HT and dopamine (DA) transporters, monoamine oxidases A and B, and various receptor types (α- and β-adrenoreceptors, DA-, GABA-, adenosine-, muscarinic-, nicotinic-, histamine-, glutamate-, benzodiazepine-, and sigma-receptors) as well as sodium, potassium and calcium channels, among which are the pharmacological targets accounting notably for the adverse side effects of tricyclics (de Bodinat et al., 2010; Guardiola-Lemaitre et al., 2014). Accordingly, agomelatine has the potential to become a novel antidepressant drug of clinical interest for a better treatment of neuropathic pain in humans.

However, to date, only animal models of somatic neuropathic pain have been tested for assessing the potential anti-allodynic/anti-hyperalgesic effects of agomelatine. In particular, clear-cut results were obtained in rats suffering from neuropathic pain triggered by sciatic nerve ligation or oxaliplatin treatment, or associated with streptozotocin-induced diabetes, in which agomelatine was reported to significantly reduce mechanical and cold allodynia/hyperalgesia at hindpaw level (M’Dahoma et al., 2015; Uçel et al., 2015; Aydin et al., 2016; Chenaf et al., 2017).

Extensive studies have shown that neuropathic pain at cephalic level has specificities compared to extra-cephalic/somatic level and responds differently to alleviating drugs both in animals (Latrémolière et al., 2008; Castro et al., 2017) and in humans (Finnerup et al., 2015, 2016; Rehm et al., 2018). In particular morphine and tricyclic antidepressants appeared to be much more potent against somatic versus cephalic neuropathic pain (Idänpään-Heikkila and Guilbaud, 1999; Michot et al., 2012b), whereas, in contrast, triptans and CGRP antagonists efficiently alleviate cephalic neuropathic pain but are almost completely inactive against somatic neuropathic pain (Kayser et al., 2002, 2011; Michot et al., 2012a, 2014). These data led us to assess whether or not the anti-neuropathic pain properties of agomelatine evidenced at somatic (hindpaw) level could also be demonstrated at cephalic level. To this goal, we investigated possible changes by this drug of mechanical allodynia induced by infraorbital nerve ligation compared to sciatic nerve ligation in rats. Furthermore, we also tested the association of agomelatine + gabapentin as previous studies showed that combined treatment with an antidepressant and an anticonvulsant drug could produce synergistic alleviating effects in neuropathic pain models (Gilron et al., 2013; Gilron, 2014; Kremer et al., 2016). Finally, because neurochemical and electrophysiological investigations showed that agomelatine treatment may affect both NA and 5-HT neurotransmissions (Millan et al., 2003; Chenu et al., 2013), experiments with NA and 5-HT receptor antagonists were performed in order to assess whether NA- and/or 5-HT-related mechanisms could mediate, at least in part, the alleviating action of agomelatine in neuropathic rats.

## Materials and Methods

### Animals

Male Sprague–Dawley rats, weighing 175–200 g (7–8 weeks-old) on arrival in the laboratory, were purchased from Charles River Breeding Center (69210 L’Arbresle, France). They were housed under standard environmental conditions (22 ± 1°C, 60% relative humidity, 12:12 h light–dark cycle, lights on at 7:00 am), on cell bed chips Maxi (SAFE, 89290 Augy, France), with complete diet for rats (ref. 105, SAFE) and tap water available *ad libitum*. Before surgery, rats were housed 5 per cage (40 cm × 40 cm, 20 cm high) and allowed to habituate to the housing facilities without any handling for at least 1 week before being used. After surgery, all efforts were made to minimize suffering. In particular, nerve-ligated rats were housed under the very same conditions, except that each cage was for only three operated rats, so as to avoid as much as possible allodynic contacts between them. Animals were thoroughly examined each day, and in case of any sign of abnormal physiological alterations or suffering, they were immediately sacrificed by a lethal dose of pentobarbital (150 mg/kg i.p.). In all cases, experiments were performed in strict conformity with the Ethical Guidelines of the Committee for Research and Ethical Issues of the International Association for the Study of Pain (Zimmermann, 1983) and the recommendations of the Ethical Committee of the French Ministry of Research and High Education (articles R.214-124, R.214-125). Accordingly, the national (French) Committee for Animal Care and Use for Scientific Research specifically approved the study (registration nb.01296.01; official authorization B75-116 to M.H., 31 December 2012).

### Chronic Constriction Injury to the Infraorbital Nerve (CCI-ION)

Rats were anesthetized with pentobarbital (50 mg/kg i.p.), and unilateral CCI-ION was performed under direct visual control using a Zeiss microscope (10–25×) essentially as described by Vos et al. (1994). Briefly, the head was fixed in a Horsley-Clarke stereotaxic frame and a midline scalp incision was made, exposing skull and nasal bone. The edge of the orbit, formed by the maxillary, frontal, lachrymal, and zygomatic bones, was dissected free on the right side. The orbital contents were then gently deflected to give access to the infraorbital nerve, which was dissected free at its most rostral extent in the orbital cavity, just caudal to the infraorbital foramen. Only 5 mm of the nerve could be freed (Vos et al., 1994; Kayser et al., 2002), providing the space for placement of two silk (5–0) ligations tied loosely (with about 2 mm spacing) around it. To obtain the desired degree of constriction, the ligations were tightened up to reducing the diameter of the nerve by a just noticeable amount to retard, but not interrupt, epineurial circulation (Bennett and Xie, 1988). Finally, scalp incision was closed using silk sutures (4–0). In sham-operated control rats, the ION was exposed using the same procedure, but was not ligated.

### Chronic Constriction Injury to the Sciatic Nerve (CCI-SN)

Rats were anesthetized as above, and the common sciatic nerve was exposed on the right side. Using a headband magnifier (2.75×), four silk (5–0) ligations were tied loosely with about 1 mm spacing, proximally to the sciatic trifurcation (Bennett and Xie, 1988). Thereafter the muscle and skin were sewed using silk sutures (4–0). In sham-operated control animals, the same surgery was performed, but the nerve was not ligated.

After both CCI-ION and CCI-SN surgeries, rats were gently put on a warming pad until recovery from anesthesia and then returned to their home cages (3 animals per cage).

### Pharmacological Treatments

All treatments were administered acutely via the i.p. route 14–16 days after surgery, when hyperalgesia and allodynia remained stable at their maximal levels in both CCI-SN and CCI-ION rats (Latrémolière et al., 2008; M’Dahoma et al., 2015). Behavioral tests were performed just before injection, and then at 30 min intervals for 4 h after injection, always by an experienced person blind to treatment groups.

When gabapentin was co-administered with agomelatine, melatonin, RS 127445, SB 206553 or SB 242084, injections of each drugs’duo were made within less than 1 min. Other pharmacological treatments (ICI 118551, idazoxan, propranolol, S22153) were administered 30 min before injection of the combination of agomelatine + gabapentin. Doses were chosen from relevant data in the literature: agomelatine, 10, 20 and 45 mg/kg (M’Dahoma et al., 2015; Chenaf et al., 2017), melatonin, 45 mg/kg (Chenaf et al., 2017), gabapentin, 50 mg/kg (Chenaf et al., 2017), β-adrenoreceptor antagonists: propranolol, 10 mg/kg (Aydin et al., 2016; Chenaf et al., 2017) and ICI 118551, 2 mg/kg (Bilski et al., 1983; Yalcin et al., 2009), α_2_-adrenoreceptor antagonist: idazoxan, 2 mg/kg (Aubel et al., 2004; Chenaf et al., 2017), MT_1_/MT_2_ receptor antagonist: S22153, 20 mg/kg (Kopp et al., 1999; Papp et al., 2003), 5-HT_2B_/5-HT_2C_ receptor antagonist: SB 206553, 2.5 and 10 mg/kg (Kennett et al., 1996), 5-HT_2B_ receptor antagonists: RS127445, 5 and 20 mg/kg (Bonhaus et al., 1999), and LY 266097, 0.63 mg/kg (Cussac et al., 2002), 5-HT_2C_ receptor antagonist: SB 242084: 2.5 and 10 mg/kg (Kennett et al., 1997). Sham-operated control rats received only the corresponding vehicles (0.9% NaCl for gabapentin and 1% hydroxyethylcellulose, HEC, in water for all other drugs) in the same volume (1 ml/kg) and at the very same times as active drugs in treated rats. All drug formulations were prepared freshly, just before administration.

### Behavioral Testing

#### Von Frey Filaments Test in CCI-ION Rats

Before any stimulation session, each rat freely explored the observation cage (35 cm × 20 cm, 15 cm high) and the testing environments for a 2 h acclimatization period. Then mechanical sensitivity was determined with a graded series of ten von Frey filaments (Bioseb, 13127 Vitrolles, France) producing a bending force of 0.07, 0.16, 0.40, 0.60, 1.00, 2.00, 4.00, 6.00, 8.00, and 10.00 g, respectively. The stimuli were applied within the ION territory (vibrissal pad), three times with each filament (with at least 3 s intervals, allowing the rat to return in its initial resting state) on the nerve-injured side. For each session, von Frey filaments were tested in increasing force order up to the one producing a response (Vos et al., 1994; Kayser et al., 2010). Behavioral nocifensive response consisted of either (1) a brisk withdrawal reaction: the rat pulled briskly backward; (2) an escape/attack: the rat avoided further contact with the filament either passively by moving its body away from the stimulating object to assume a crouching position against cage wall, sometimes with the head buried under the body, or actively by attacking the stimulating object, making biting and grabbing movements; or (3) asymmetric face grooming: the rat displayed an uninterrupted series of at least 3 face-wash strokes directed to the stimulated facial area, often preceded by brisk withdrawal reaction. As explained in previous reports (see Vos et al., 1994; Kayser et al., 2002, 2010; Latrémolière et al., 2008), the minimal force filament causing at least one among these aversive responses to at least 2 out of the 3 filament applications allowed determination of the mechanical response threshold. The 10.00 g filament was the cut-off threshold (no tissue-injury occurred with this pressing force).

#### Von Frey Filaments Test in CCI-SN Rats

Before testing, each rat was habituated for 2 h on a metal mesh floor, under a small plastic cage (35 cm × 20 cm, 15 cm high), and mechanical sensitivity was then determined with a graded series of eight von Frey filaments, producing a bending force of 4, 6, 8, 10, 12, 15, 26, and 60 g, respectively. The stimuli were applied within the SN territory (lateral plantar surface of the ipsilateral hind paw). Each filament was tested three times on the nerve-injured side in increasing order up to trigger a nocifensive reaction consisting of a brisk paw withdrawal and/or an escape attempt (see Kayser et al., 2010). As for the CCI-ION rats, a time interval of at least 3 s allowed the rat to recover its initial resting state between two filament applications. The minimal force filament for which animals presented a nocifensive response to at least 2 out of the 3 stimulations allowed determination of the mechanical response threshold (Latrémolière et al., 2008; Michot et al., 2012a, 2013). The 60 g filament was chosen as the cut-off threshold.

### Chemicals

Agomelatine, S22153 (*N*-[2-(5-ethylbenzo[*b*]thiophen-3yl)ethyl] acetamide), and HEC were from Servier (Suresnes, France). Gabapentin was from Sequoia Research Products (Pangbourne, United Kingdom). Melatonin, propranolol and idazoxan were from Sigma-Aldrich (Saint-Quentin-Fallavier, France). ICI 118551 [(±)-erythro-(S^∗^,S^∗^)-1-[2,3-(dihydro-7-methyl-1*H*-inden-4-yl)oxy]-3-[(1-methylethyl)amino]-2-butanol], LY 266097 (1-(2-chloro-3,4-dimethoxybenzyl)-6-methyl-1,2,3,4-tetrahydro-9*H*-pyrido[3,4-b]indole) and SB 242084 (6-chloro-5-methyl-*N*-[6-(2-methylpyridin-3-yloxy)pyridin-3-yl]indoline-1-carboxamide) were from Tocris (Lille, France). SB 206553 (5-methyl-*N*-(3-pyridyl)-1,2,3,5-tetrahydrobenzo[1,2-b:4,5-b′]dipyrrole-1-carboxamide) was from ABCAM (Paris, France) and RS 127445 (4-(4-fluoro-1-naphthalenyl)-6-(1-methylethyl)-2-pyrimidinamine) was from Selleck Chemicals (Munich, Germany).

### Statistics

Results are expressed as mean ± SEM. Repeated measures’ analysis of variance (one way ANOVA) followed by Dunnett’s test was conducted to assess the effects of drugs over time. Treatment effect for each drug was determined by comparing values after injection to control value (time 0, just prior to injection) using a one-way ANOVA followed by a Dunnett’s *post hoc* test. Areas under the time-course curves (AUC) were calculated using the trapezoidal rule, and statistical significance of differences in AUC values corresponding to various treatment groups was assessed using a one-way ANOVA followed by a Tukey’s *post hoc* test. For all tests, the significance level was set at *P* < 0.05.

## Results

**FIGURE 1:**
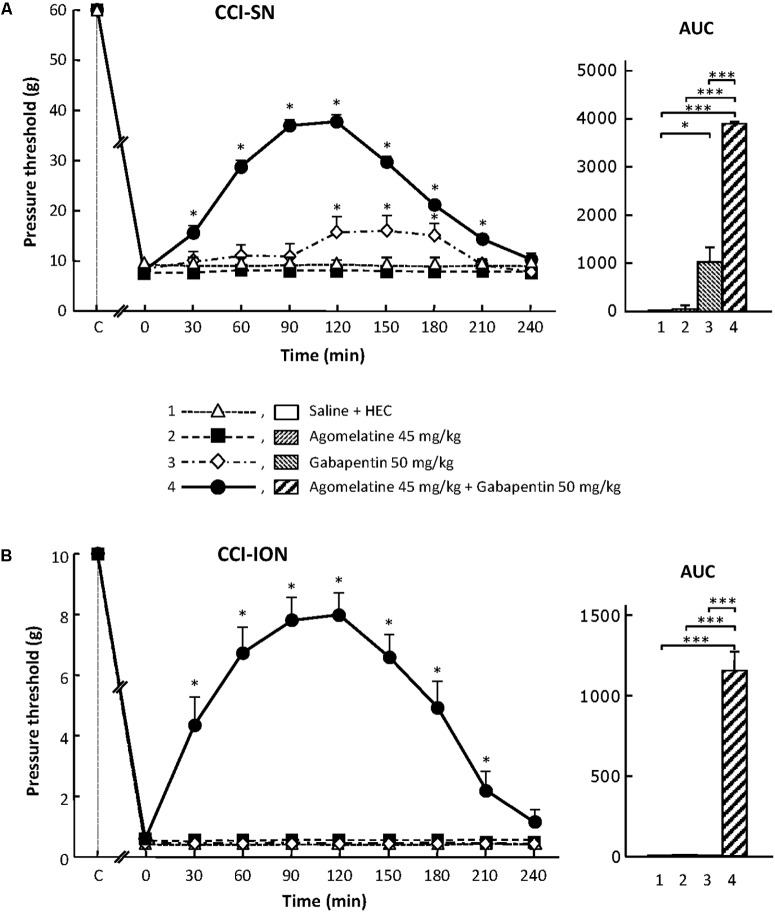
Effects of agomelatine, gabapentin and their combination on mechanical allodynia in CCI-SN **(A)** and CCI-ION **(B)** rats. Left panels: Agomelatine (45 mg/kg), gabapentin (50 mg/kg), agomelatine + gabapentin and/or respective vehicles (saline, HEC) were injected i.p. 2 weeks after nerve ligation. Pressure threshold values (as g) were determined using von Frey filaments applied onto the ipsilateral hindpaw (A-CCI-SN) or vibrissal pad (B-CCI-ION) at various times after injections (abscissa). Each point is the mean ± SEM of *n* independent determinations. ^∗^*P* < 0.05, compared with pressure threshold values determined just prior to drug injection (0 on abscissa), one way ANOVA with repeated measures, Dunnett’s test. C on abscissa: intact healthy rats before surgery. Right panels*:* AUC values calculated from the respective time-course curves: (1) saline + HEC [*n* = 25 **(A)**, *n* = 13 **(B)**]; (2) agomelatine + saline (*n* = 7/5); (3) gabapentin + HEC (*n* = 9/6); (4) agomelatine + gabapentin (*n* = 40/28). A- CCI-SN : one way ANOVA [*F*(3,77) = 92.39, *P* < 0.0001] followed by Tukey’s test (^∗^*P* < 0.05, ^∗∗∗^*P* < 0.001); B- CCI-ION: one way ANOVA [*F*(3,48) = 24.19, *P* < 0.0001] followed by Tukey’s test (^∗∗∗^*P* < 0.001).

In sham-operated animals, as in intact healthy rats, a mechanical pressure of up to 60 g (cut-off threshold) had to be applied through von Frey filament onto a hindpaw in order to trigger a response (hindpaw withdrawal) in about half of them. In contrast, a pressure as low as 6 g was enough to trigger hindpaw withdrawal in CCI-SN rats (**Figure 1A**), indicating the occurrence of marked mechanical allodynia after sciatic nerve ligation.

Similarly, mechanical pressure with von Frey filament of up to 10 g (cut-off threshold) had to be applied onto the vibrissae territory to trigger some behavioral reaction (head movement to escape filament pressure) in about half of control (naive or sham-operated) rats. In contrast, 2 weeks after CCI-ION, a mechanical pressure of only 0.2–0.4 g, or even less for some rats, was enough to trigger a brisk withdrawal of the head or attack toward the filament, indicating the occurrence of marked mechanical allodynia in the territory of the ligated infraorbital nerve (**Figure 1B**).

### Agomelatine Exerts an Antiallodynic Effect Only When Combined With Gabapentin in CCI-SN and CCI-ION Rats

In both CCI-SN and CCI-ION rats, no change in pressure threshold value to trigger nocifensive reactions was observed for up to 4 h after acute administration of agomelatine at 10, 20, or 45 mg/kg i.p. (**Figure 1** and data not shown). On the other hand, acute treatment with gabapentin at the dose of 50 mg/kg i.p. produced a modest but significant increase in pressure threshold value to trigger ipsilateral hindpaw withdrawal in CCI-SN rats (**Figure 1A**). In contrast, gabapentin at the same dose was totally ineffective to reduce mechanical allodynia in CCI-ION rats (**Figure 1B**).

Although each drug alone was either completely ineffective or only partly effective, the combined administration of agomelatine (45 mg/kg i.p.) plus gabapentin (50 mg/kg i.p.), which affected neither spontaneous global behavior nor locomotor activity (not shown), produced large increases in pressure threshold values to trigger nocifensive reactions in both CCI-SN (**Figure 1A**) and CCI-ION (**Figure 1B**) rats. In both groups, significant changes were observed as soon as 30 min post-injections, reached maximal amplitudes at 90–120 min, and then progressively vanished so that respective threshold values did not differ from those in vehicle-treated nerve ligated rats on the 4th hour post-injections (**Figures 1A,B**). Interestingly, treatment with ‘agomelatine + gabapentin’ maximally increased respective pressure threshold values up to fourfold compared to the control value in saline-treated CCI-SN rats (**Figure 1A**) and up to 20-fold compared to the control value in saline-treated CCI-ION rats (**Figure 1B**), indicating an apparent anti-allodynic effect relatively more pronounced in CCI-ION rats than in CCI-SN rats.

### Are MT_1_/MT_2_ Receptors Implicated in the Antiallodynic Effects of Agomelatine + Gabapentin?

#### Effects of Melatonin Alone or Combined With Gabapentin in CCI-SN and CCI-ION Rats

In order to assess the possible contribution of MT_1_/MT_2_ receptor stimulation in the anti-allodynic effect of the combination ‘agomelatine + gabapentin,’ we investigated whether melatonin alone or combined with gabapentin exerted some effect in nerve-ligated rats. In both CCI-SN and CCI-ION rats, neither the administration of melatonin at 45 mg/kg i.p. nor that of the combination ‘melatonin (45 mg/kg i.p.) + gabapentin (50 mg/kg i.p.)’ significantly affected pressure threshold values for up to 4 h post-treatment (data not shown).

#### Effect of MT_1_/MT_2_ Receptors Blockade by S22153 on the Anti-allodynic Effect of the Combination ‘Agomelatine + Gabapentin’ in CCI-SN and CCI-ION Rats

**FIGURE 2:**
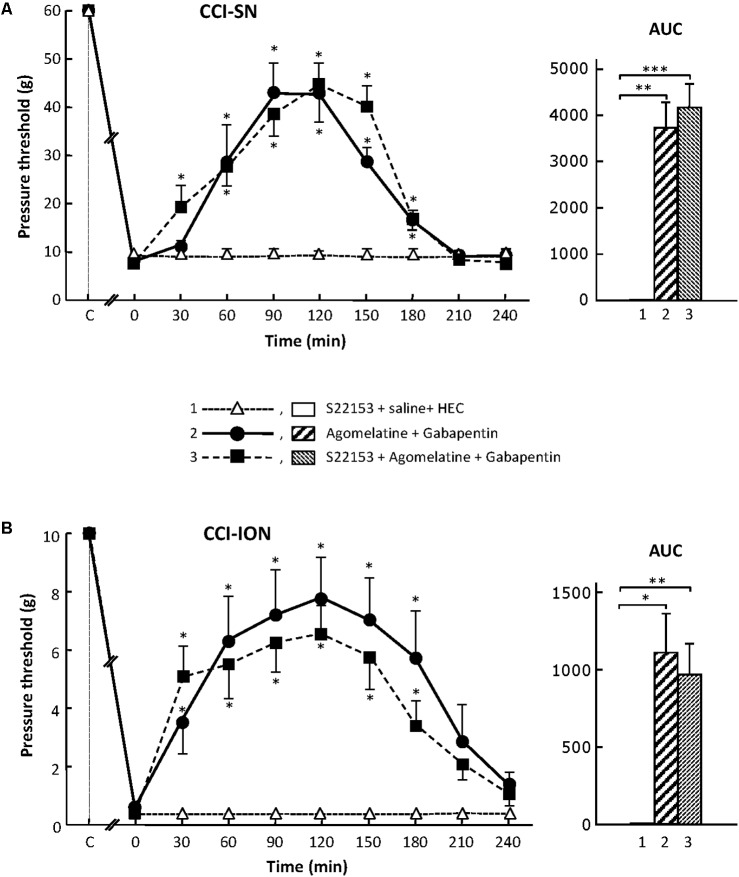
Effect of pretreatment with S22153 on mechanical allodynia inhibition by ‘agomelatine + gabapentin’ in CCI-SN **(A)** and CCI-ION **(B)** rats. Left panels: (1) S22153 (20 mg/kg) was injected i.p. 30 min before administration of agomelatine (45 mg/kg i.p.) + gabapentin (50 mg/kg i.p.) – at time 0 on abscissa – in rats whose right SN **(A)** or ION **(B)** nerve had been ligated 2 weeks before. Pressure threshold values (as g) were determined as described in the legend to **Figure 1**. Each point is the mean ± SEM of *n* independent determinations. ^∗^*P* < 0.05 compared with pressure threshold values determined just prior to drug injection, one way ANOVA with repeated measures, Dunnett’s test. C on abscissa: intact healthy rats before surgery. In both CCI-SN **(A)** and CCI-ION **(B)** rats, ‘saline+HEC’-treated groups were also included, but, for the sake of clarity, corresponding data are not represented as they superimposed with those obtained in rats treated with S22153 plus these vehicles. Right panels: AUC values calculated from the respective time-course curves: (1) S22153 + saline + HEC [*n* = 5 **(A)**, *n* = 5 **(B)**]; (2) agomelatine + gabapentin (*n* = 6/7); (3) S22153 + agomelatine + gabapentin (*n* = 11/14). A- CCI-SN : one way ANOVA [*F*(2,18) = 17.40, *P* < 0.0001] followed by Tukey’s test (^∗∗^*P* < 0.01, ^∗∗∗^*P* < 0.001); B- CCI-ION : one way ANOVA [*F*(2,25) = 5.913, *P* = 0.0079] followed by Tukey’s test (^∗^*P* < 0.05, ^∗∗^*P* < 0.01).

In order to further assess the possible implication of MT_1_/MT_2_ receptors in the anti-allodynic effect of the combination ‘agomelatine + gabapentin’ in nerve ligated rats, we investigated whether pretreatment with the specific MT_1_/MT_2_ receptor antagonist, S22153 (Kopp et al., 1999), could reduce the increases in pressure threshold values induced by this drug combination in both CCI-SN and CCI-ION rats. As illustrated in **Figure 2**, S22153 (20 mg/kg i.p.) on its own did not produce any change in pressure threshold values in CCI-SN and CCI-ION rats as well as in respective sham controls (not shown). In addition, the time-course as well as the amplitude of the anti-allodynic effects of ‘agomelatine + gabapentin’ did not differ whether or not this drug combination was preceded by pretreatment with S22153 thirty min before in both CCI-SN (**Figure 2A**) and CCI-ION (**Figure 2B**) rats.

Altogether, these results showed that the activation of MT_1_/MT_2_ receptors by agomelatine is not sufficient (if concerned) to account for the anti-allodynic properties of the combination ‘agomelatine + gabapentin.’

### Are 5-HT_2B_ and/or 5-HT_2C_ Receptors Implicated in the Antiallodynic Effects of ‘Agomelatine + Gabapentin’?

Because the other two molecular targets of agomelatine are 5-HT_2B_ and 5-HT_2C_ receptors, at which the drug acts as an antagonist (Millan et al., 2003), we then investigated whether 5-HT_2B_ and/or 5-HT_2C_ receptor antagonists on their own or in combination with gabapentin (50 mg/kg i.p.) could mimic the anti-allodynic effects of ‘agomelatine + gabapentin’ in both CCI-SN and CCI-ION rats.

**FIGURE 3:**
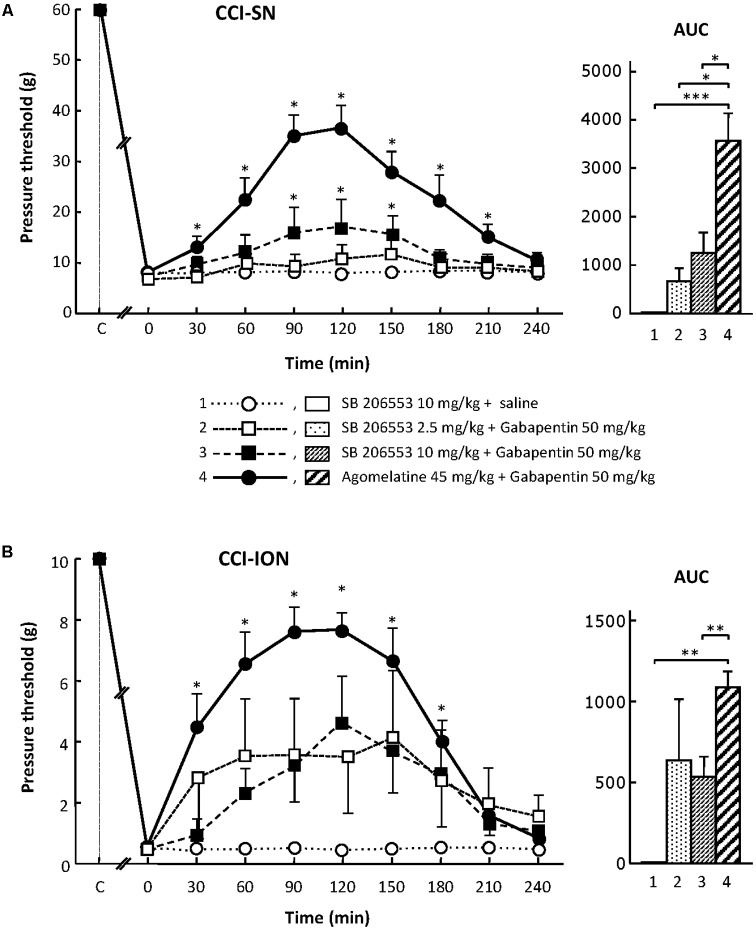
Effects of SB 206553 alone or co-administered with gabapentin on mechanical allodynia in CCI-SN **(A)** and CCI-ION **(B)** rats. Comparison with the anti-allodynic effect of ‘agomelatine + gabapentin.’ Left panels: SB 206553 (2.5 or 10 mg/kg) + saline, SB 206553 (2.5 or 10 mg/kg) + gabapentin (50 mg/kg), agomelatine (45 mg/kg) + gabapentin and/or respective vehicles (saline, HEC) were injected i.p. 2 weeks after nerve ligation. Pressure threshold values (as g) were determined as described in the legend to **Figure 1**. Each point is the mean ± SEM of *n* independent determinations. ^∗^*P* < 0.05, compared with pressure threshold values determined just prior to drug injection (0 on abscissa), one way ANOVA with repeated measures, Dunnett’s test. C on abscissa: intact healthy rats before surgery. In both CCI-SN **(A)** and CCI-ION **(B)** rats, ‘saline+HEC’-treated groups were also included, but, for the sake of clarity, corresponding data are not represented as they superimposed with those obtained in rats treated with S206553 plus saline. Right panels: AUC values calculated from the respective time-course curves: (1) SB 206553 + saline [*n* = 6 **(A)**, *n* = 6 **(B)**]; (2) SB 206553 (2.5 mg/kg) + gabapentin (*n* = 6/6); (3) SB 206553 (10 mg/kg) + gabapentin (*n* = 6/13); (4) agomelatine + gabapentin (*n* = 8/6). A- CCI-SN: one way ANOVA [*F*(3,22) = 10.97, *P* < 0.0001] followed by Tukey’s test (^∗^*P* < 0.05, ^∗∗∗^ *P* < 0.001); B- CCI-ION: one way ANOVA [*F*(3,27) = 6.972, *P* = 0.0013] followed by Tukey’s test (^∗∗^*P* < 0.01).

When tested alone, SB 206553 (2.5 or 10 mg/kg i.p.), a mixed 5-HT_2B_/5-HT_2C_ receptor antagonist (Kennett et al., 1996), did not significantly modify nerve-ligation-induced decreases in pressure threshold values, for at least 4 h after administration, in both CCI-SN and CCI-ION rats (**Figure 3** and data not shown). As shown in **Figures 3A,B**, co-treatment with gabapentin (50 mg/kg i.p.) produced some increase in pressure threshold values in both nerve-ligated groups. However, at its maximum, the change evoked by the combination ‘SB 206553 + gabapentin’ reached only 30–50% of the effect produced by agomelatine (45 mg/kg i.p.) + gabapentin (50 mg/kg i.p.), and was statistically significant only in CCI-SN rats (**Figures 3A,B**). Rather than increasing the number of rats treated with the non-selective 5-HT_2B_/5-HT_2C_ receptor antagonist SB 206553 to possibly reach statistical significance also in CCI-ION rats, we chose to further investigate 5-HT_2_ receptor implication by using antagonists acting selectively at only one 5-HT_2_ receptor subtype.

**FIGURE 4:**
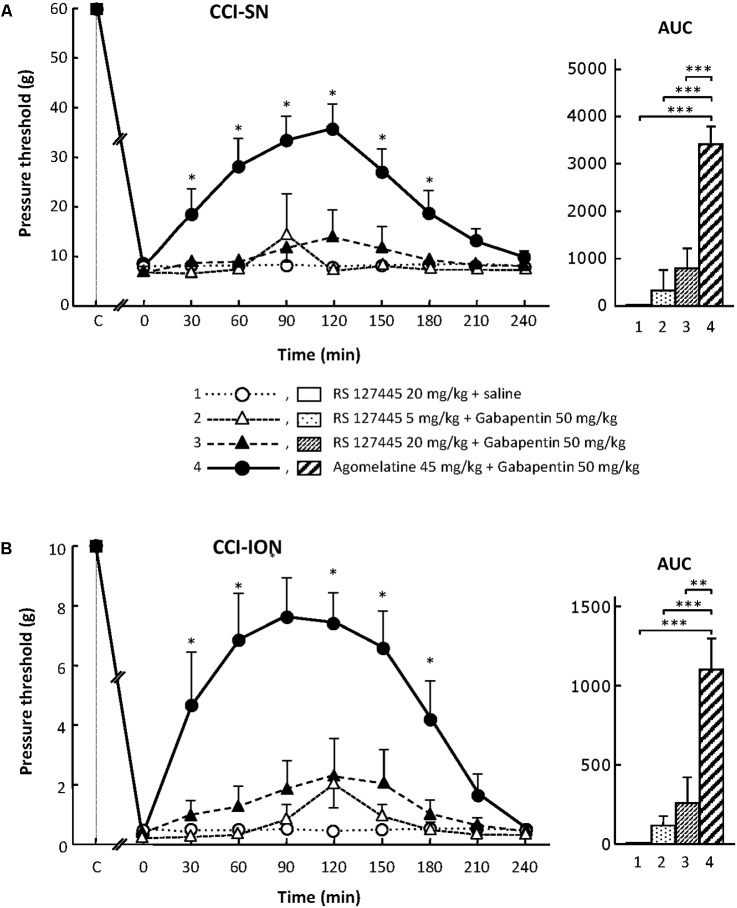
Effects of RS 127445 alone or co-administered with gabapentin on mechanical allodynia in CCI-SN **(A)** and CCI-ION **(B)** rats. Comparison with the anti-allodynic effect of ‘agomelatine + gabapentin.’ Left panels: RS 127445 (20 mg/kg), RS 127445 (5 or 20 mg/kg) + gabapentin (50 mg/kg), agomelatine (45 mg/kg) + gabapentin and/or respective vehicles (saline, HEC; not shown) were injected i.p. 2 weeks after nerve ligation. Pressure threshold values (as g) were determined as described in the legend to **Figure 1**. Each point is the mean ± SEM of *n* independent determinations. ^∗^*P* < 0.05, compared with pressure threshold values determined just prior to drug injection (0 on abscissa), one way ANOVA with repeated measures, Dunnett’s test. C on abscissa: intact healthy rats before surgery. In both CCI-SN **(A)** and CCI-ION **(B)** rats, ‘saline+HEC’-treated groups were also included, but, for the sake of clarity, corresponding data are not represented as they superimposed with those obtained in rats treated with RS 127445 plus saline. Right panels*:* AUC values calculated from the respective time-course curves: (1) RS 127445 (20 mg/kg) + saline [*n* = 6 **(A)**, *n* = 5 **(B)**]; (2) RS 127445 (5 mg/kg) + gabapentin (*n* = 6/7); (3) RS 127445 (20 mg/kg) + gabapentin (*n* = 6/8); (4) agomelatine + gabapentin (*n* = 7/7). A- CCI-SN : one way ANOVA [*F*(3,21) = 25.20, *P* < 0.0001] followed by Tukey’s test (^∗∗∗^*P* < 0.001); B- CCI-ION : one way ANOVA [*F*(3,23) = 11.86, *P* < 0.0001] followed by Tukey’s test (^∗∗^*P* < 0.01, ^∗∗∗^*P* < 0.001).

On their own, the two selective 5-HT_2B_ receptor antagonists, RS 127445 (5 or 20 mg/kg i.p., **Figure 4**) and LY 266097 (0.63 mg/kg i.p., not shown), exerted no effect on nerve ligation-induced-decrease in pressure threshold values in both CCI-SN and CCI-ION rats. Furthermore, both drugs remained essentially ineffective even when combined with gabapentin (50 mg/kg i.p.), as pressure threshold values did not significantly differ whether nerve-ligated rats were treated with vehicles, RS 127445 + gabapentin (**Figure 4**) or LY 266097 + gabapentin (data not shown).

**FIGURE 5:**
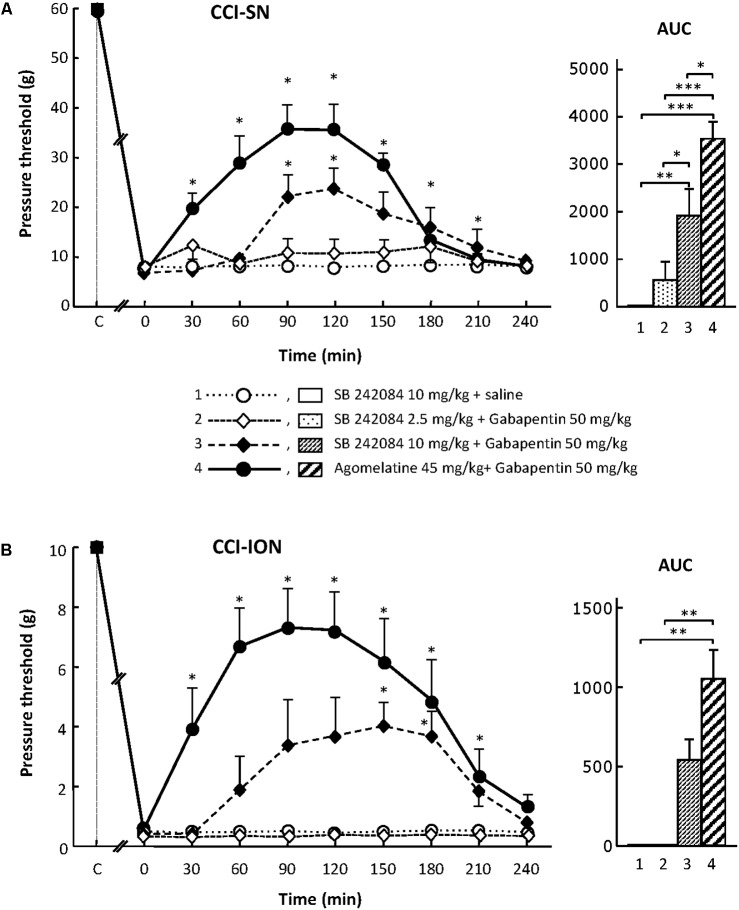
Effects of SB 242084 alone or co-administered with gabapentin on mechanical allodynia in CCI-SN **(A)** and CCI-ION **(B)** rats. Comparison with the anti-allodynic effect of ‘agomelatine + gabapentin.’Left panels: SB 242084 (10 mg/kg), SB 242084 (2.5 or 10 mg/kg) + gabapentin (50 mg/kg), agomelatine (45 mg/kg) + gabapentin, and/or respective vehicles (saline, HEC; not shown) were injected i.p. 2 weeks after nerve ligation. Pressure threshold values (as g) were determined as described in the legend to **Figure 1**. Each point is the mean ± SEM of n independent determinations. ^∗^*P* < 0.05, compared with pressure threshold values determined just prior to drug injection (0 on abscissa), one way ANOVA with repeated measures, Dunnett’s test. C on abscissa: intact healthy rats before surgery. In both CCI-SN **(A)** and CCI-ION **(B)** rats, ‘saline+HEC’-treated groups were also included, but, for the sake of clarity, corresponding data are not represented as they superimposed with those obtained in rats treated with SB 242084 plus saline. Right panels: AUC values calculated from the respective time-course curves: (1) SB 242084 (10 mg/kg) + saline [*n* = 7 **(A)**, *n* = 5 **(B)**]; (2) SB 242084 (2.5 mg/kg) + gabapentin (*n* = 8/6); (3) SB 242084 (10 mg/kg) + gabapentin (*n* = 10/8); (4) agomelatine + gabapentin (*n* = 7/7). A- CCI-SN : one way ANOVA [*F*(3,28) = 16.76, *P* < 0.0001] followed by Tukey’s test (^∗^*P* < 0.05, ^∗∗^*P* < 0.01, ^∗∗∗^*P* < 0.001); B- CCI-ION: one way ANOVA [*F*(3,22) = 7.98, *P* = 0.0009] followed by Tukey’s test (^∗∗^*P* < 0.01).

For assessing the possible implication of 5-HT_2C_ receptors, we used their selective antagonist SB 242084 at appropriate dosage: 2.5 or 10 mg/kg i.p. (Kennett et al., 1997) either alone or co-administered with gabapentin at 50 mg/kg i.p. On its own, SB 242084 was ineffective at these doses, but the association of the 10 mg/kg dose with gabapentin exerted a significant anti-allodynic effect in both CCI-SN and CCI-ION rats (**Figures 5A,B**). As compared to that evoked by agomelatine + gabapentin, the resulting increase in pressure threshold values was about half, corresponding to approximately 30% recovery of control values found in vehicle-treated sham operated animals (**Figures 5A,B**).

Altogether, these data suggest that the 5-HT_2C_ receptor subtype plays a role in the anti-allodynic properties of agomelatine co-administered with gabapentin.

### Both α- and β-Adrenoreceptor Activation Is Required for the Anti-allodynic Effect of the Association ‘Agomelatine + Gabapentin’ in Nerve Ligated Rats

Because (i) the blockade of 5-HT_2C_ receptors by agomelatine may result in a stimulatory effect on NA release in some CNS areas (Millan et al., 2003), and (ii) noradrenergic neurotransmission exerts an inhibitory influence on neuropathic pain (Pertovaara, 2006), experiments were designed to assess the participation of adrenoreceptors in the anti-allodynic effects of ‘agomelatine + gabapentin’ in CCI-SN and CCI-ION rats.

#### Partial Blockade by Idazoxan of the Anti-allodynic Effect of the Association ‘Agomelatine + Gabapentin’ in CCI-SN and CCI-ION Rats

**FIGURE 6:**
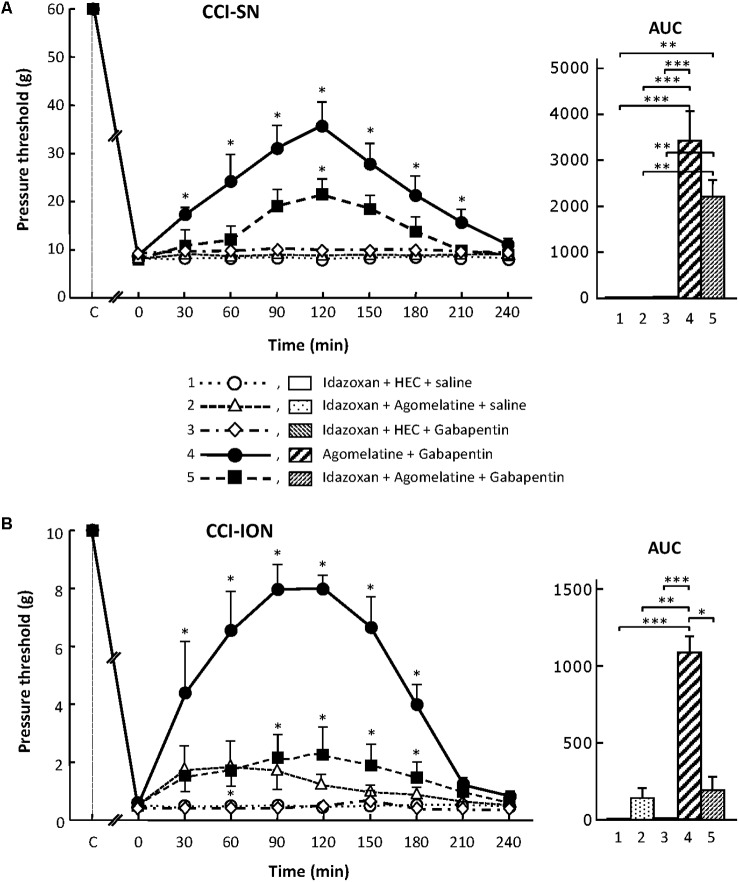
Effects of idazoxan alone or co-administered with agomelatine, gabapentin or ‘agomelatine + gabapentin’ on mechanical allodynia in CCI-SN **(A)** and CCI-ION **(B)** rats. Left panels: Idazoxan (2 mg/kg i.p.) or its vehicle (saline) was administered 30 min before other treatments in rats whose right SN **(A)** or ION **(B)** had been ligated 2 weeks before. Five different treatment groups were made: (1) idazoxan + HEC + saline, (2) idazoxan + agomelatine (45 mg/kg) + saline, (3) idazoxan + HEC + gabapentin (50 mg/kg), (4) agomelatine + gabapentin, (5) idazoxan + agomelatine + gabapentin. Pressure threshold values (as g) were determined as described in the legend to **Figure 1**. Each point is the mean ± SEM of independent determinations in *n* rats for each condition. ^∗^*P* < 0.05, compared with pressure threshold values determined just prior to treatments (0 on abscissa), one way ANOVA with repeated measures, Dunnett’s test. C on abscissa: intact healthy rats before surgery. In both CCI-SN **(A)** and CCI-ION **(B)** rats, ‘saline+HEC’-treated groups were also included, but, for the sake of clarity, corresponding data are not represented as they superimposed with those obtained in rats treated with idazoxan + HEC + saline. Right panels: AUC values calculated from the respective time-course curves: (1) idazoxan + HEC + saline [*n* = 7 **(A)**, *n* = 5 **(B)**]; (2) idazoxan + agomelatine + saline (*n* = 6/6); (3) idazoxan + HEC + gabapentin (*n* = 6/5); (4) agomelatine + gabapentin (*n* = 7/6); 5 = idazoxan + agomelatine + gabapentin (*n* = 7/14). A- CCI-SN : one way ANOVA [*F*(4,28) = 16.33, *P* < 0.0001] followed by Tukey’s test (^∗∗^*P* < 0.01, ^∗∗∗^*P* < 0.001); B- CCI-ION : one way ANOVA [*F*(4,35) = 8.267, *P* < 0.0001] followed by Tukey’s test (^∗^*P* < 0.05, ^∗∗^*P* < 0.01, ^∗∗∗^*P* < 0.001).

Although treatment with the α_2_-adrenoreceptor antagonist idazoxan, at the dose of 2 mg/kg i.p. (to block peripheral and central α_2_-adrenoreceptors; Aubel et al., 2004) did not, on its own, modify pressure threshold values in both control (sham-operated) and nerve-ligated rats, this treatment significantly reduced the anti-allodynic effect of ‘agomelatine + gabapentin’ when performed 30 min before administration of the latter drug combination (**Figures 6A,B**). Meanwhile, idazoxan injected with agomelatine or gabapentin alone exerted no effects on CCI-SN-induced allodynia (**Figure 6A**), and only minor non-significant effects (idazoxan + agomelatine) on CCI-ION-induced allodynia (**Figure 6B**). Further comparison between AUC values determined for the two models of neuropathic pain shows that idazoxan-induced reduction in mechanical allodynia was larger in CCI-ION rats (-83%) than in CCI-SN rats (-60%). This difference suggests that α_2_-adrenoreceptor activation probably contributed to the anti-allodynic effect of ‘agomelatine + gabapentin’ much less in CCI-SN compared to CCI-ION rats. This led to assess whether β-adrenoreceptors might also be differentially involved in the effect of ‘agomelatine + gabapentin’ in the two groups of neuropathic rats. Accordingly, β-adrenoreceptor antagonists were tested.

#### Inhibition by β-Adrenoreceptor Blockade of the Anti-allodynic Effect of the Combination ‘Agomelatine + Gabapentin’ in CCI-SN and CCI-ION Rats

##### Effects of propranolol

**FIGURE 7:**
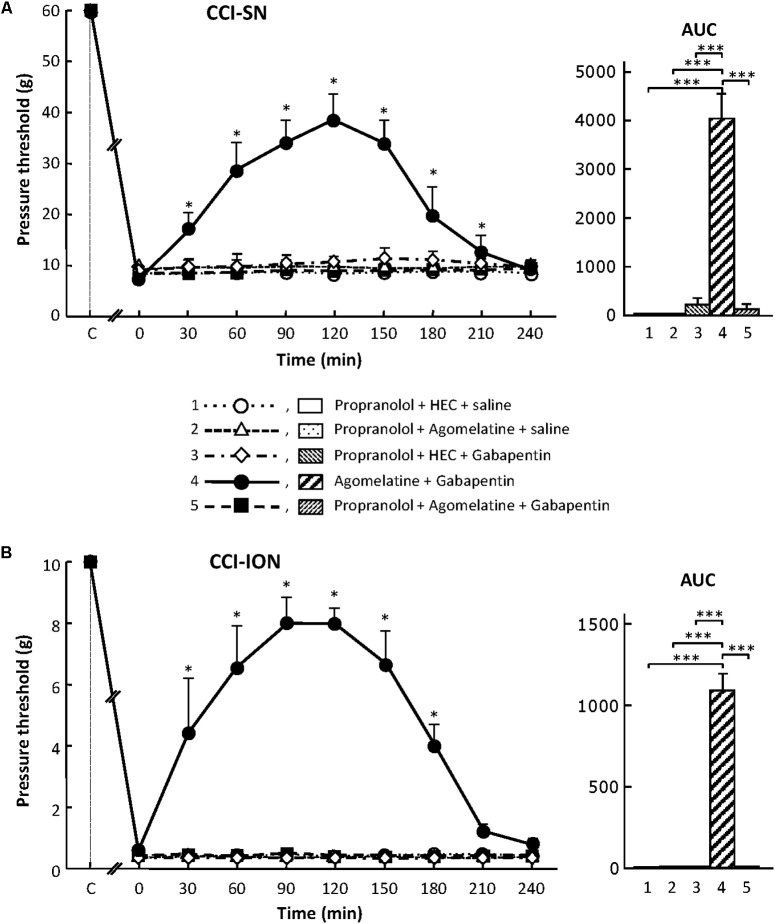
Effects of propranolol alone or co-administered with agomelatine, gabapentin or ‘agomelatine + gabapentin’ on mechanical allodynia in CCI-SN **(A)** and CCI-ION **(B)** rats. Left panels: Propranolol (10 mg/kg i.p.) or its vehicle (saline) was administered 30 min before other treatments in rats whose right SN **(A)** or ION **(B)** had been ligated 2 weeks before. Five different treatment groups were made: (1) propranolol + HEC + saline, (2) propranolol + agomelatine (45 mg/kg) + saline, (3) propranolol + HEC + gabapentin (50 mg/kg), (4) agomelatine + gabapentin, (5) propranolol + agomelatine + gabapentin. Pressure threshold values (as g) were determined as described in the legend to **Figure 1**. Each point is the mean ± SEM of independent determinations in *n* rats for each condition. ^∗^*P* < 0.05, compared with pressure threshold values determined just prior to treatments (0 on abscissa), one way ANOVA with repeated measures, Dunnett’s test. C on abscissa: intact healthy rats before surgery. In both CCI-SN **(A)** and CCI-ION **(B)** rats, ‘saline+HEC’-treated groups were also included, but, for the sake of clarity, corresponding data are not represented as they superimposed with those obtained in rats treated with propranolol + HEC + saline. Right panels: AUC values calculated from the respective time-course curves: (1) propranolol + HEC + saline [*n* = 5 **(A)**/5 **(B)**]; (2) propranolol + agomelatine + saline (*n* = 5/5); (3) propranolol + HEC + gabapentin (*n* = 5/5); (4) agomelatine + gabapentin (*n* = 8/7); 5 = propranolol + agomelatine + gabapentin (*n* = 14/11). A- CCI-SN : one way ANOVA [*F*(4,32) = 73.51, *P* < 0.0001] followed by Tukey’s test (^∗∗∗^*P* < 0.001); B- CCI-ION: one way ANOVA [*F*(4,28) = 25.39, *P* < 0.0001] followed by Tukey’s test (^∗∗∗^*P* < 0.001).

As shown in **Figure 7**, propranolol, at the dose of 10 mg/kg i.p. to block β_1_/β_2_- adrenoreceptors (Aydin et al., 2016), did not modify CCI-induced decreases in pressure threshold values in both CCI-SN and CCI-ION rats. Co-treatments with propranolol + agomelatine or gabapentin alone were also ineffective (**Figure 7**). However, administration of propranolol 30 min before injections of ‘agomelatine + gabapentin’ totally prevented the anti-allodynic effect of the latter drugs’ combination in both CCI-SN (**Figure 7A**) and CCI-ION (**Figure 7B**) rats.

##### Effects of ICI 118551

**FIGURE 8:**
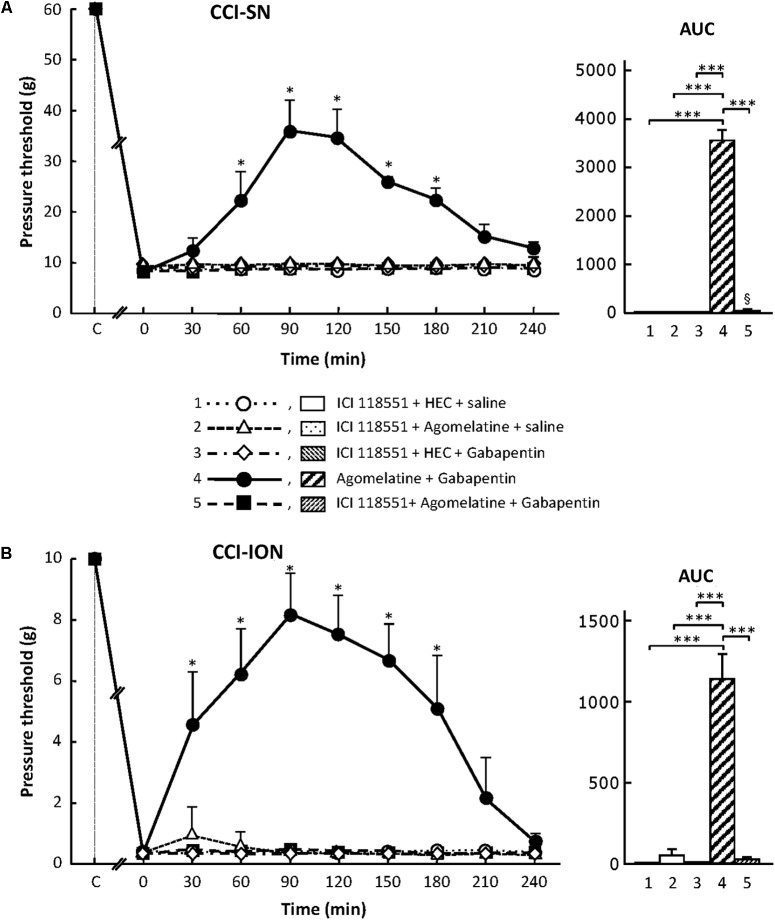
Effects of ICI 118551 alone or co-administered with agomelatine, gabapentin or ‘agomelatine + gabapentin’ on mechanical allodynia in CCI-SN **(A)** or CCI-ION **(B)** rats. Left panels: ICI 118551 (5 mg/kg i.p.) or its vehicle (saline) was administered 30 min before other treatments in rats whose right SN **(A)** or ION **(B)** had been ligated 2 weeks before. Five different treatment groups were made: (1) ICI 118551 + HEC + saline, (2) ICI 118551 + agomelatine (45 mg/kg) + saline, (3) ICI 118551 + HEC + gabapentin (50 mg/kg), (4) agomelatine + gabapentin, (5) ICI 118551 + agomelatine + gabapentin. Pressure threshold values (as g) were determined as described in the legend to **Figure 1**. Each point is the mean ± SEM of independent determinations in *n* rats for each condition. ^∗^*P* < 0.05, compared with pressure threshold values determined just prior to drug injection, one way ANOVA with repeated measures, Dunnett’s test. C on abscissa: intact healthy rats before surgery. In both CCI-SN **(A)** and CCI-ION **(B)** rats, ‘saline+HEC’-treated groups were also included, but, for the sake of clarity, corresponding data are not represented as they superimposed with those obtained in rats treated with ICI 118551 + HEC + saline. Right panels: AUC values calculated from the respective time-course curves: 1 = ICI 118551 + HEC + saline [*n* = 5 **(A)**, *n* = 5 **(B)**]; 2 = ICI 118551 + agomelatine + saline (*n* = 5/5); 3 = ICI 118551 + HEC + gabapentin (*n* = 5/6); 4 = agomelatine + gabapentin (*n* = 6/7); 5 = ICI 118551 + agomelatine + gabapentin (*n* = 13/7). A- CCI-SN : one way ANOVA [*F*(4,29) = 310.9, *P* < 0.0001] followed by Tukey’s test (^∗∗∗^*P* < 0.001); B- CCI-ION : one way ANOVA [*F*(4,25) = 39.35, *P* < 0.0001] followed by Tukey’s test (^∗∗∗^*P* < 0.001).

Treatment with the specific β_2_-adrenoreceptor antagonist, ICI 118551 (5 mg/kg i.p.; Bilski et al., 1983; Yalcin et al., 2009), alone or combined with either agomelatine or gabapentin had no effect on nerve ligation-induced decrease in pressure threshold values in both CCI-SN and CCI-ION rats (**Figure 8**). However, administration of ICI 118551 thirty min before ‘agomelatine + gabapentin’ completely prevented the anti-allodynic effect of the latter drugs’ combination in CCI-SN (**Figure 8A**) as well as in CCI-ION (**Figure 8B**) rats.

Altogether, these results show that α_2_- and β_2_-adrenoreceptors are implicated in the anti-allodynic effects of the combination ‘agomelatine + gabapentin’ in both CCI-SN and CCI-ION rats.

## Discussion

To date, pharmacological treatments of neuropathic pain implicate antidepressants and anticonvulsants in first-line, although these drugs have a limited efficacy and are endowed with poorly tolerated side effects (Gilron, 2014; Finnerup et al., 2015, 2016). Furthermore, their efficacy may vary from one type of neuropathic pain to another, with trigeminal neuropathic pain generally less responsive than other extra-cephalic neuropathic pain. This has also been verified in validated animal models such as those achieved by nerve ligation. Thus, whereas tricyclic antidepressants are quite potent to reduce mechanical hyperalgesia and allodynia caused by ligation of the sciatic nerve for instance (Micó et al., 2006), these drugs have been reported to be essentially ineffective against neuropathic pain-like behaviors generated by ligation of the infraorbital nerve (Idänpään-Heikkila and Guilbaud, 1999; Michot et al., 2012b).

Interestingly, the recently developed antidepressant, agomelatine, whose mechanism of action markedly differs from those of other antidepressants currently used to alleviate neuropathic pain, e.g., tricyclics and mixed inhibitors of 5-HT and NA reuptake (Micó et al., 2006), has also been found to possess anti-hyperalgesic properties in several rat models of extra-cephalic neuropathic pain (M’Dahoma et al., 2015; Uçel et al., 2015; Aydin et al., 2016; Chenaf et al., 2017). As effective neuropathic pain alleviating treatments are even more needed against cephalic (trigeminal) pain (Finnerup et al., 2015, 2016), whether or not agomelatine is also effective at cephalic level was herein investigated using a validated model of this type of pain, the CCI-ION rat (Vos et al., 1994). Neuropathic-like pain was assessed by quantifying nerve ligation-induced mechanical allodynia using the von Frey filament test under conditions allowing comparison between CCI-ION and CCI-SN rats (Latrémolière et al., 2008).

As it rapidly appeared that, under acute treatment conditions, agomelatine was ineffective on its own, we investigated whether it could develop anti-allodynic effects when combined with an anticonvulsant, namely gabapentin. Indeed, an abundant literature has shown that combined treatments with an antidepressant and an anticonvulsant most often produce synergistic effects, with much better alleviation of neuropathic pain than that expected from the sum of the effects expected from each drug alone (Tomic et al., 2010; Miyazaki and Yamamoto, 2012; Gilron et al., 2013; Miranda et al., 2015). In both CCI-SN and CCI-ION rats, we found here that although gabapentin was administered at a dose, 50 mg/kg i.p., which produces no sedative effect and only minor, or even no, anti-allodynic effect (Christensen et al., 2001; Patel et al., 2001; De Vry et al., 2004; Vanelderen et al., 2013), co-treatment with agomelatine + gabapentin effectively produced marked anti-allodynic effects. Interestingly, as previously noted for agomelatine alone in the same dose range (Kasap and Can, 2016; Chenaf et al., 2017) as that used in our studies, no changes in global behavior and locomotor activity were observed after acute administration of the combination of the latter drug + gabapentin (50 mg/kg i.p.; data not shown), in agreement with Chenaf et al. (2017).

Accordingly, such a striking synergy between agomelatine and gabapentin to suppress nerve ligation-induced mechanical allodynia could not be accounted for by some non-specific effects, but revealed an interesting novel pharmacological potentiality to alleviate neuropathic pain. This led us to investigate further the neurobiological mechanisms underlying the anti-allodynic effect of ‘agomelatine + gabapentin’ using appropriate pharmacological paradigms. Because antidepressants act mainly at monoaminergic neurotransmission (Micó et al., 2006), and gabapentin can activate NA neurotransmission at spinal sites involved in pain control mechanisms (Takeuchi et al., 2007), we focused most of our investigations on the well known NA and 5-HT pain modulatory systems (Millan, 2002; Pertovaara, 2006), by using respective antagonists, to assess the potential implication of these monoaminergic systems in the effects of ‘agomelatine + gabapentin.’

However, MT_1_/MT_2_ receptors had also to be considered because literature data support the idea that MT, the endogenous ligand of these agomelatine targets, might be endowed with alleviating properties in neuropathic pain models (Ambriz-Tututi et al., 2009), probably through its anti-inflammatory and anti-oxidant actions (Mayo et al., 2005), also shared by agomelatine (Molteni et al., 2013). More precisely, investigations using selective ligands and mutant mice pointed to the MT_2_ receptor as the molecular target responsible for neuropathic pain alleviating MT action (Yu et al., 2000; Srinivasan et al., 2012; Lopez-Canul et al., 2015). However, in agreement with previous reports (Ulugol et al., 2006; Ambriz-Tututi and Granados-Soto, 2007; Wang et al., 2009), in the dose range used in our study, MT was devoid of any anti-allodynic effect in nerve lesioned rats. Furthermore, MT was unable to reproduce the capacity of agomelatine to decrease supersensitivity to mechanical stimulation in both CCI-SN and CCI-ION rats co-treated with gabapentin. On the other hand, pretreatment with the mixed MT_1_/MT_2_ receptor antagonist, S22153, at the dose of 20 mg/kg i.p. to block peripheral and central MT receptors (Kopp et al., 1999), did not significantly interfere with the anti-allodynic effect of ‘agomelatine + gabapentin’ in both CCI-SN and CCI-ION rats. Altogether, our data support the idea that agomelatine interaction with MT receptors *per se* does not account for the capacity of the drug combination to decrease mechanical supersensitivity in nerve lesioned rats. In contrast, Chenaf et al. (2017) recently reported that the anti-hyperalgesic effect of agomelatine (45 mg/kg i.p.) assessed using the Randall-Selitto test in CCI-SN rats could be mimicked by MT (45 mg/kg i.p.) and prevented by the MT_1_/MT_2_ receptor antagonist S22153 (20 mg/kg i.p.). These discrepancies further illustrate that mechanical allodynia, assessed with von Frey filaments in our study, and mechanical hyperalgesia, assessed in the study of Chenaf et al. (2017), are underlain through different neurobiological mechanisms, and are differentially responsive to analgesic drugs (Sandkühler, 2009; Barrot, 2012; Michot et al., 2014).

To assess whether the antagonistic action of agomelatine at 5-HT_2B_ and 5-HT_2C_ receptors (Millan et al., 2003) might play a role in the anti-allodynic effect of co-treatment with this drug and gabapentin, we investigated whether antagonists at these 5-HT_2_ receptor subtypes could mimic this effect, either alone or co-administered with gabapentin. The first series of data obtained with the mixed 5-HT_2B_/5-HT_2C_ receptor antagonist SB 206553 suggested that at least one of these 5-HT_2_ receptor subtypes could be involved because a clear-cut reduction in nerve ligation-induced mechanical allodynia was observed in both CCI-SN and CCI-ION rats treated with this ligand combined with gabapentin. Subsequent investigations with the selective 5-HT_2B_ antagonists RS 127445 and LY 266097 (not shown) on the one hand and the selective 5-HT_2C_ receptor antagonist SB 242084 on the other hand, at appropriate doses to block these respective receptors (Kennett et al., 1997; Bonhaus et al., 1999; Auclair et al., 2010), showed that only the latter drug produced a significant decrease in nerve ligation-induced mechanical allodynia. However, at the dose used, high enough to block completely 5-HT_2C_ receptors (Kennett et al., 1997), and administered together with gabapentin, SB 242084 mimicked only partially the anti-allodynic effect of ‘agomelatine + gabapentin.’ Accordingly, the latter effect was probably not underlain solely by the antagonistic action of agomelatine at 5-HT_2C_ receptors *per se*. Indeed, the latter receptors physically interact with MT_2_ receptors, and MT_2_/5-HT_2C_ heteromers were found to amplify the 5-HT_2C_-mediated Gq/phospholipase C response to 5-HT and cause MT-induced unidirectional transactivation of the 5-HT_2C_ protomer in such receptor complex (Kamal et al., 2015). Therefore, such cross-talk between MT_2_ and 5-HT_2C_ protomers within the MT_2_/5-HT_2C_ receptor complex probably results in modified (synergistic) responses to concomitant activation of each protomer by agomelatine (see Ferré et al., 2014), which might account for its unique pharmacological properties (Tardito et al., 2012; Guardiola-Lemaitre et al., 2014).

As the neuropathic pain alleviating properties of tricyclics and mixed NA/5-HT reuptake inhibitors were shown to be mediated, at least partly, through a facilitation of noradrenergic neurotransmission (Micó et al., 2006), we then investigated whether the anti-allodynic effect of ‘agomelatine + gabapentin’ could also be dependent on adrenoreceptor activation. Indeed, Millan et al. (2003) demonstrated that acute treatment with agomelatine enhances NA release in the rat brain, and we postulated that this effect might also be extended to bulbo-spinal NA neurotransmission, which plays a key role in pain control mechanisms (Pertovaara, 2006). Furthermore, gabapentin was also shown to activate bulbo-spinal NA neurotransmission, and increase NA turnover at spinal level (Takeuchi et al., 2007). Therefore, combined actions of agomelatine and gabapentin might produce a marked increase in spinal NA neurotransmission, underlying the anti-allodynic action of co-treatment with these two drugs. In line with this hypothesis, we found that administration of the α_2_-adrenoreceptor antagonist idazoxan significantly reduced the anti-allodynic effect of agomelatine + gabapentin, as expected of a key role of this receptor subtype in NA-dependent antinociceptive mechanisms (Sullivan et al., 1987; Özdogan et al., 2004; Pertovaara, 2006). Similarly, the anti-hyperalgesic effect of agomelatine alone in CCI-SN rats was recently found to be markedly reduced by pre-treatment with idazoxan (Chenaf et al., 2017), suggesting that NA mechanisms inhibiting both mechanical allodynia and hyperalgesia caused by CCI-SN implicate α_2_-adrenergic receptors. In contrast, discrepant results were found with propranolol since Chenaf et al. (2017) reported that this β-adrenoreceptor antagonist does not affect agomelatine-induced anti-hyperalgesia whereas we did find a clear inhibition of the anti-allodynic effect of ‘agomelatine + gabapentin’ in both CCI-SN and CCI-ION rats pre-treated with propranolol. However, it would be hazardous to conclude that β-adrenoreceptors contribute to the anti-allodynic effect but not the anti-hyperalgesic effect of agomelatine because Aydin et al. (2016) recently found that propranolol (10 mg/kg p.o.) efficiently abrogates the anti-hyperalgesic effect of agomelatine in diabetic rats suffering from neuropathy. With regard to nerve ligation-induced allodynia, we found that the effects of propranolol were mimicked by the selective β_2_-adrenoreceptor antagonist ICI 118551, suggesting that this subtype of β-adrenoreceptors is especially implicated in NA-mediated control of neuropathic pain by ‘agomelatine + gabapentin.’ Similar data have been reported for other antidepressants, namely the tricyclic desipramine and selective or mixed NA reuptake inhibitors such as reboxetine and venlafaxine, by Yalcin et al. (2009) who reported that their anti-allodynic action was also inhibited by ICI 118551 in another model of neuropathic pain in mice. In line with these findings, Zhang et al. (2016) recently pointed out that the β_2_-adrenoreceptor, but not the β_1_-adrenoreceptor, also mediates the inhibitory effect of spinal NA on neuropathic pain caused by partial sciatic nerve ligation.

Altogether, these data strongly support the idea that NA neurotransmission, involving probably bulbo-spinal noradrenergic projections (Micó et al., 2006; Kremer et al., 2016), is a common target of antidepressants endowed with anti-hyperalgesic and/or anti-allodynic properties, including agomelatine co-administered with gabapentin. Similar dual contribution of both α_2_- and β_2_-adrenoreceptors in NA-mediated pain control mechanisms as that found in our study has been reported in models of diabetic neuropathy (Aydin et al., 2016) and collagen-induced inflammatory pain (Park et al., 2013), supporting the idea that activation of these two receptor subtypes, probably at the spinal level (Pertovaara, 2006; Kremer et al., 2016), plays key roles in endogenous pain control mechanisms. Although further studies are needed to elucidate the respective roles of α_2_- and β_2_-adrenoreceptors in NA-mediated mechanisms underlying the anti-allodynic effects of ‘agomelatine + gabapentin,’ one can speculate that α_2_-adrenoreceptor-mediated action might occur presynaptically, through an inhibition of nociceptive transmitter release from primary afferent fibers (Millan, 2002), whereas β_2_-adrenoreceptor-mediated action would involve an inhibition of “post-synaptic” glial activation processes within the dorsal horn (see Zhang et al., 2016).

Regarding the first goal of our investigations, i.e., comparison of anti-allodynic effects in CCI-SN versus CCI-ION rats, ‘agomelatine + gabapentin’ markedly differs from other pain alleviating drugs, especially tricyclic antidepressants (Idänpään-Heikkila and Guilbaud, 1999), because this drug combination was effective in both models. In this regard, it closely resembles tapentadol, a mixed μ opioid receptor agonist and NA reuptake inhibitor, which exerts potent anti-allodynic effects at both cephalic and extra-cephalic levels (Michot et al., 2013). As ‘agomelatine + gabapentin,’ like tapentadol, also acts through NA neurotransmission, it can be asked whether a concomitant action through opioidergic mechanisms might contribute to its effective anti-allodynic effects in both CCI-SN and CCI-ION rats. Indeed, Kasap and Can (2016) recently demonstrated that opioid receptor activation underlays at least part of the antinociceptive effect of agomelatine, thereby providing support to the hypothesis that mixed opioid-NA mechanisms might account for the remarkable anti-allodynic potency of ‘agomelatine + gabapentin’ at both cephalic and extra-cephalic levels. Interestingly, as found here with ‘agomelatine + gabapentin,’ combination of melatonin + dextromethorphan, a potent NMDA receptor antagonist, was also reported to produce anti-allodynic effects at doses ineffective for each drug administered alone (Wang et al., 2009), suggesting that agomelatine-induced inhibition of glutamatergic neurotransmission (see Reagan et al., 2012) might also contribute to the synergistic effects of both drugs combinations.

Finally, although CCI-SN and CCI-ION models both responded to the anti-allodynic effect of ‘agomelatine + gabapentin,’ our data show that the latter “cephalic” pain model seemed to be more responsive than the “extra cephalic” model, suggesting that allodynia associated with trigeminal pain and migraine might be especially targeted by this drug combination, and indeed preliminary clinical evidence in humans support this view (Guglielmo et al., 2013; Plasencia-Garcia et al., 2015). Interestingly, like for the antidepressant action of agomelatine alone in humans (Kasper and Hamon, 2009), no tolerance to the anti-allodynic action of ‘agomelatine + gabapentin’ was observed in a preliminary study where CCI-SN rats had been treated daily for 2 weeks with the drug combination (Dabala et al., 2015). Although these data are promising, the long term effectiveness of chronic treatment with ‘agomelatine + gabapentin’ will have to be demonstrated in double-blind studies before considering this drug combination as a novel treatment for neuropathic pain alleviation in humans.

## Author Contributions

SM, CG, EM, MH, and SB designed the study. SM, MP, ED, HP, and SB performed the experiments and analyzed the data. SM, MH, and SB wrote the first draft of the manuscript. All authors contributed to and approved the final manuscript.

## Conflict of Interest Statement

CG and EM are employed by Institut de Recherches Internationales Servier. The other authors declare that the research was conducted in the absence of any commercial or financial relationships that could be construed as a potential conflict of interest.
